# Mortality trends in deaths attributed to adverse drug reactions with cardiovascular disease listed among multiple causes in the United States, 1999–2020

**DOI:** 10.3389/fcvm.2026.1857514

**Published:** 2026-10-01

**Authors:** Xing Guo, Kun Zheng, Lingying Su, Hong Zhu

**Affiliations:** 1 Department of Pharmacy, The Affiliated Huaian No.1 People’s Hospital of Nanjing Medical University, Huaian, Jiangsu, China; 2 Department of Neurology, The Affiliated Huaian No.1 People's Hospital of Nanjing Medical University, Huaian, Jiangsu, China; 3 Department of Technology Cooperation and Transformation, The Affiliated Huaian No.1 People’s Hospital of Nanjing Medical University, Huaian, Jiangsu, China

**Keywords:** adverse drug reactions, anticoagulants, cardiovascular disease, mortality, pharmacovigilance

## Abstract

**Background:**

Adverse drug reactions (ADRs) represent a significant cause of preventable mortality and continue to pose a major challenge to medication safety. However, national temporal and demographic patterns of deaths in which an ADR was assigned as the underlying cause and cardiovascular disease (CVD) was also listed on the death certificate remain poorly characterized.

**Methods:**

We conducted a death certificate-based descriptive study using the CDC WONDER Multiple Cause of Death database from 1999 to 2020. Included records were deaths in which an ADR-related ICD-10 code (Y40–Y59) was assigned as the underlying cause of death and a CVD code (I00–I99) was listed among the multiple causes of death. Age-adjusted mortality rates (AAMRs) per 100,000 population were calculated using the 2000 U.S. standard population. Temporal trends were assessed with Joinpoint regression, estimating annual percent change (APC) and average APC (AAPC). Mortality patterns were further analyzed by age, sex, race, region, and urbanization level, and drug categories recorded on the included death certificates were summarized descriptively.

**Results:**

From 1999 to 2020, 3,976 CVD-listed ADR deaths were identified, corresponding to an overall AAMR of 0.057 per 100,000 U.S. population. The AAMR of these deaths increased significantly over the study period (AAPC = 4.755%, 95% CI: 3.513–6.428; *P* < 0.001). Mortality burden increased with age, particularly among individuals aged 75 years and older. Males exhibited higher mortality rates than females, and significant disparities were evident by race and geography, with the highest burden observed in Black individuals and rural populations. Drug-specific analysis showed that unspecified drugs were recorded in 1,398 deaths (35.2%). Anticoagulants were the most frequently recorded specified drug category (*n* = 948; 23.8% of all included deaths), particularly among decedents with cerebrovascular disease.

**Conclusion:**

CVD-listed ADR mortality in the United States rose significantly from 1999 to 2020. The burden was most pronounced in older adults and disproportionately high among males, Black individuals, and rural residents. Anticoagulants were the most frequently recorded specified drug category. These descriptive findings identify population-level patterns that warrant confirmation through studies incorporating medical records, prescribing data, and case-level clinical adjudication.

## 1 Introduction

Adverse drug reactions (ADRs) pose a significant challenge to global public health, as they not only contribute to increased morbidity and hospitalization but can also directly result in death in severe cases, creating a substantial healthcare burden (1). In 2022, the U.S. Food and Drug Administration Adverse Event Reporting System received more than 1.25 million serious adverse-event reports, including nearly 175,000 reports involving death (2). Despite advances in pharmacovigilance, spontaneous reporting systems continue to suffer from underreporting, complicating efforts to capture the full scope of ADRs (3). Fatal ADRs, in particular, have garnered significant attention due to their irreversible and severe outcomes, with events such as anaphylactic shock posing an immediate life-threatening risk (4). While these events remain relatively infrequent, they are associated with high case-fatality rates. Thus, a thorough investigation into the characteristics of fatal ADRs, identification of high-risk drugs, and the development of effective prevention and management strategies are crucial to enhancing medication safety and improving patient outcomes.

Patients with cardiovascular diseases (CVDs) are particularly vulnerable to fatal ADRs due to their frequent exposure to chronic multidrug therapy and the common presence of advanced age, frailty, and a high burden of comorbidities (5, 6). These factors not only elevate the risk of drug–drug interactions, inappropriate prescribing, and severe adverse events, but also diminish physiological reserve, reducing patients' ability to withstand acute drug-related injuries (7, 8). Furthermore, when ADRs occur in individuals with pre-existing cardiovascular instability, such as impaired cardiac function, arrhythmia susceptibility, or vascular vulnerability, clinical deterioration may be more pronounced and rapidly progress to death (5, 9). Given the global burden of CVD and the widespread use of high-risk medications in this population, fatal ADRs among CVD patients represent a significant, yet insufficiently explored, public health issue that may be preventable (5, 10).

The Centers for Disease Control and Prevention Wide-ranging Online Data for Epidemiologic Research (CDC WONDER) database, which provides nationwide mortality data based on U.S. death certificate records, is a valuable resource for examining long-term mortality patterns at the population level (11). Its comprehensive geographic coverage and standardized cause-of-death coding make it particularly suited for investigating temporal trends and subgroup disparities. However, national patterns of national patterns of CVD-listed ADR deaths remain poorly characterized. Therefore, using the CDC WONDER database, we aimed to characterize the number, population-level mortality rates, and temporal trends of these deaths and to summarize the most frequently assigned specified drug categories.

## 2 Materials and methods

### 2.1 Study design

This population-based observational study utilized mortality data from the CDC WONDER platform, specifically the Multiple Cause of Death database, covering the period from 1999 to 2020. The objective was to assess the burden and long-term temporal trends of CVD-listed ADR mortality in the United States.

Since all data were obtained from publicly available, de-identified sources containing no personally identifiable information, the study was exempt from institutional review board approval and informed consent requirements.

### 2.2 Data acquisition

Mortality records were retrieved from the CDC WONDER Multiple Cause of Death database using a predefined extraction strategy. Fatal ADRs were identified using the International Classification of Diseases, Tenth Revision (ICD-10) codes Y40–Y59, which correspond to drugs, medicaments, and biological substances causing adverse effects in therapeutic use. Deaths were included when an ADR-related code (Y40–Y59) was assigned as the underlying cause of death and a CVD code (I00–I99) was listed among the multiple causes of death. Throughout this manuscript, these records are referred to as “CVD-listed ADR deaths”.

To characterize population-level patterns, mortality records were further stratified by sex, race, census region, and urbanization level. Sex was categorized as male or female. Race was grouped into American Indian or Alaska Native, Black or African American, White, and Asian or Pacific Islander. Geographic region was classified into the four U.S. census regions: Northeast, Midwest, South, and West. Urbanization level was defined according to the 2013 National Center for Health Statistics Urban–Rural Classification Scheme for Counties, dichotomized as metropolitan (including large central metro, large fringe metro, medium metro, and small metro) and nonmetropolitan (including micropolitan and noncore) areas (12). These subgroup classifications were used to examine demographic and geographic differences in the population-level rates of CVD-listed ADR deaths.

In accordance with CDC WONDER reporting rules, death counts below 10 were suppressed, and mortality rates based on fewer than 20 deaths were considered unreliable and were not interpreted. Suppressed estimates are presented as dashes and were treated as unavailable.

### 2.3 Statistical analysis

Descriptive analyses were conducted to summarize the distribution of CVD-listed ADR mortality across calendar years and population subgroups. Mortality counts were extracted for each stratum, and age-adjusted mortality rates (AAMRs) per 100,000 population were calculated using the direct standardization method based on the 2000 U.S. standard population. This method enabled valid comparisons across subgroups with different age structures and over time.

Temporal trends in CVD-listed ADR mortality were analyzed using Joinpoint regression. This approach calculates the annual percent change (APC) and average APC (AAPC) in AAMRs, along with 95% confidence intervals (CIs). Joinpoint regression identifies significant changes in trends over time by fitting the most parsimonious segmented model supported by the data. A two-sided *P*-value < 0.05 was considered statistically significant. All statistical analyses and data visualizations were performed using R software (version 4.2.0; Free Software Foundation, Boston, MA, USA).

## 3 Results

### 3.1 Overall mortality burden and temporal trends

From 1999 to 2020, a total of 3,976 CVD-listed ADR deaths were identified in the United States, corresponding to an overall AAMR of 0.057 per 100,000 population. As shown in Table 1, the AAMR increased from 0.023 in 1999 to 0.074 in 2020. Joinpoint analysis showed a significant upward trend in the AAMR of CVD-listed ADR deaths, with an AAPC of 4.755% (95% CI: 3.513–6.428, *P* < 0.001) (Table 2).

**Table 1:** Age-adjusted mortality rates for CVD-listed ADR deaths, United States, 1999–2020.

	All year		1999		2020	
	Numbers	AAMR per 100,000 (95% CI)	Numbers	AAMR per 100,000 (95% CI)	Numbers	AAMR per 100,000 (95% CI)
Overall	3,976	0.057 (0.055, 0.059)	93	0.023 (0.018, 0.029)	321	0.074 (0.065, 0.082)
Sex						
Female	2,019	0.048 (0.046, 0.051)	43	0.023 (0.015, 0.034)	149	0.067 (0.056, 0.079)
Male	1,957	0.058 (0.055, 0.061)	50	0.027 (0.019, 0.039)	172	0.079 (0.066, 0.091)
Ethnicity						
American Indian	30	0.045 (0.029, 0.068)	–	–	–	–
Asian or Pacific Islander	97	0.02 (0.015, 0.026)	–	–	–	–
Black or African American	530	0.054 (0.048, 0.059)	13	–	53	0.12 (0.088, 0.16)
White	3,319	0.043 (0.041, 0.045)	76	0.023 (0.017, 0.03)	252	0.072 (0.063, 0.082)
Region						
Northeast	720	0.050 (0.046, 0.054)	17	–	60	0.073 (0.055, 0.096)
Midwest	917	0.045 (0.041, 0.048)	20	0.024 (0.014, 0.04)	54	0.052 (0.038, 0.07)
South	1,499	0.055 (0.052, 0.058)	33	0.017 (0.01, 0.027)	138	0.071 (0.058, 0.083)
West	840	0.045 (0.041, 0.048)	23	0.042 (0.025, 0.066)	69	0.068 (0.052, 0.087)
Urbanization						
Urban	3,084	0.042 (0.04, 0.043)	70	0.023 (0.017, 0.031)	261	0.072 (0.063, 0.082)
Rural	892	0.063 (0.058, 0.067)	23	0.044 (0.026, 0.07)	60	0.106(0.077, 0.141)

“–” indicates that the death count was suppressed because fewer than 10 deaths were recorded. Rates based on fewer than 20 deaths were considered statistically unreliable.

**Table 2:** Annual and average annual percentage changes in mortality rates for CVD-listed ADR deaths.

Variable	Segment	Segment start	Segment end	APC (95% CI)	*P*-value	AAPC (95% CI)	*P*-value
Total	1	1,999	2,020	4.755 (3.513, 6.428)	<0.001	4.755 (3.513, 6.428)	<0.001
Sex							
Female	1	1,999	2,020	4.442 (3.05, 6.289)	<0.001	4.442 (3.05, 6.289)	<0.001
Male	1	1,999	2,020	4.826 (3.492, 6.761)	<0.001	4.826 (3.492, 6.761)	<0.001

### 3.2 Age-specific patterns

Age-specific analyses highlighted a pronounced age gradient in CVD-listed ADR mortality. As illustrated in Figure 1A, mortality rates increased progressively with age, with the highest burden concentrated in older adults, particularly those aged 75–84 years and ≥85 years. Younger age groups contributed minimally to the overall mortality burden. Across most age strata, males exhibited higher mortality rates than females, with this sex difference being most evident in older age groups. Figure 1B further illustrates that the proportional contribution of ADR-related deaths remained dominated by individuals aged ≥75 years throughout the study period, with those aged ≥85 years showing the highest contribution.

**Figure 1:**
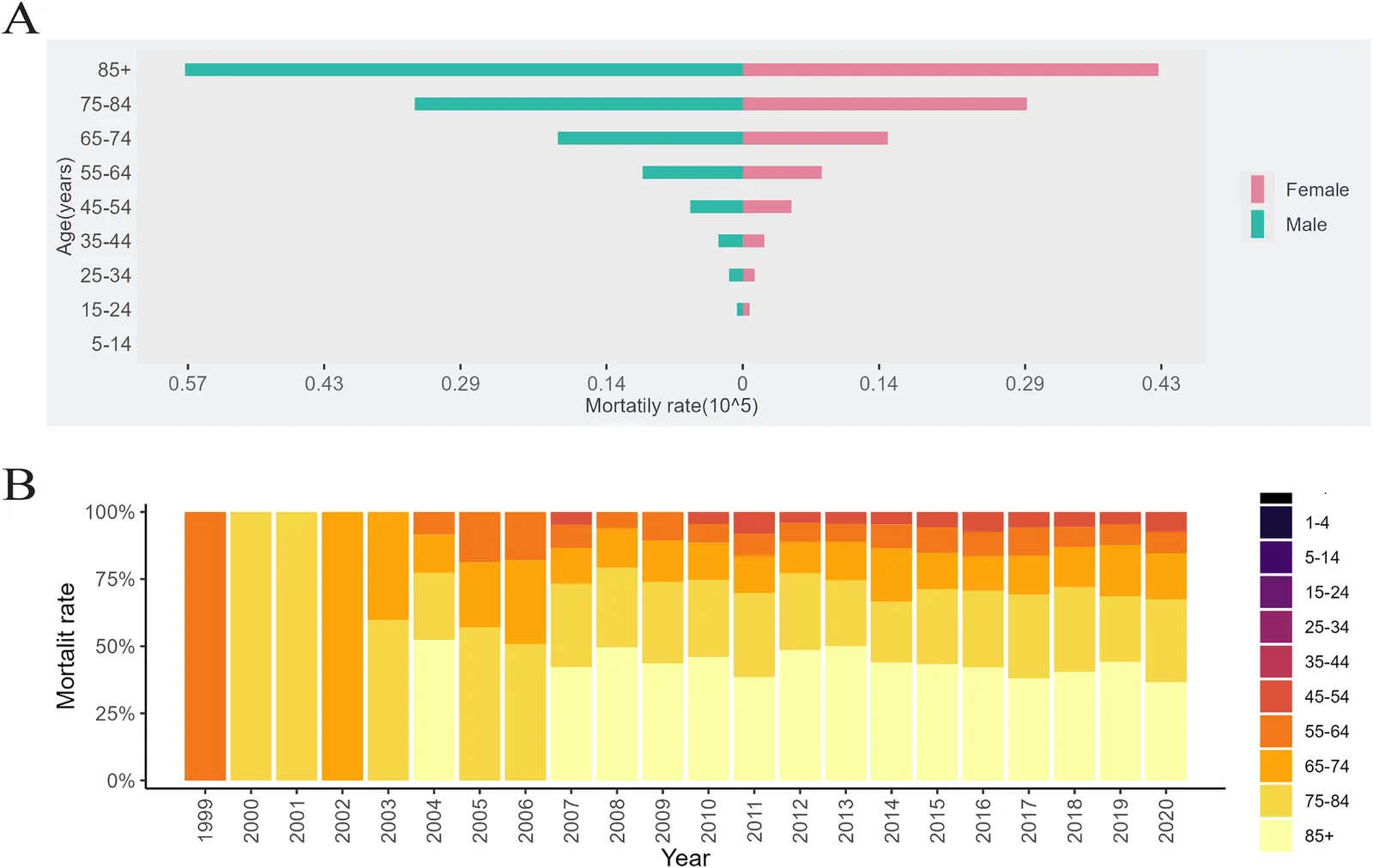
Age-specific patterns of CVD-listed ADR deaths in the United States, 1999–2020. **(A)** Age- and sex-specific mortality rates for CVD-listed ADR deaths. **(B)** Temporal trends in the age distribution of CVD-listed ADR mortality from 1999 to 2020. ADR, adverse drug reaction; CVD, cardiovascular disease.

### 3.3 Sex-specific trends

Sex-specific analyses revealed that males consistently experienced a higher burden of CVD-listed ADR mortality compared to females. The overall AAMR was 0.058 per 100,000 (95% CI: 0.055–0.061) in males, and 0.048 per 100,000 (95% CI: 0.046–0.051) in females. Figure 2 shows that mortality rates for both sexes generally increased over time, although with modest year-to-year variation. The gap between males and females remained evident across most years, with males consistently having higher AAMRs throughout the study period. Joinpoint analysis confirmed significant upward trends in both sexes, with a slightly steeper increase in males (AAPC: 4.826%, 95% CI: 3.492–6.761, *P* < 0.001) than in females (AAPC: 4.442%, 95% CI: 3.050–6.289, *P* < 0.001).

**Figure 2:**
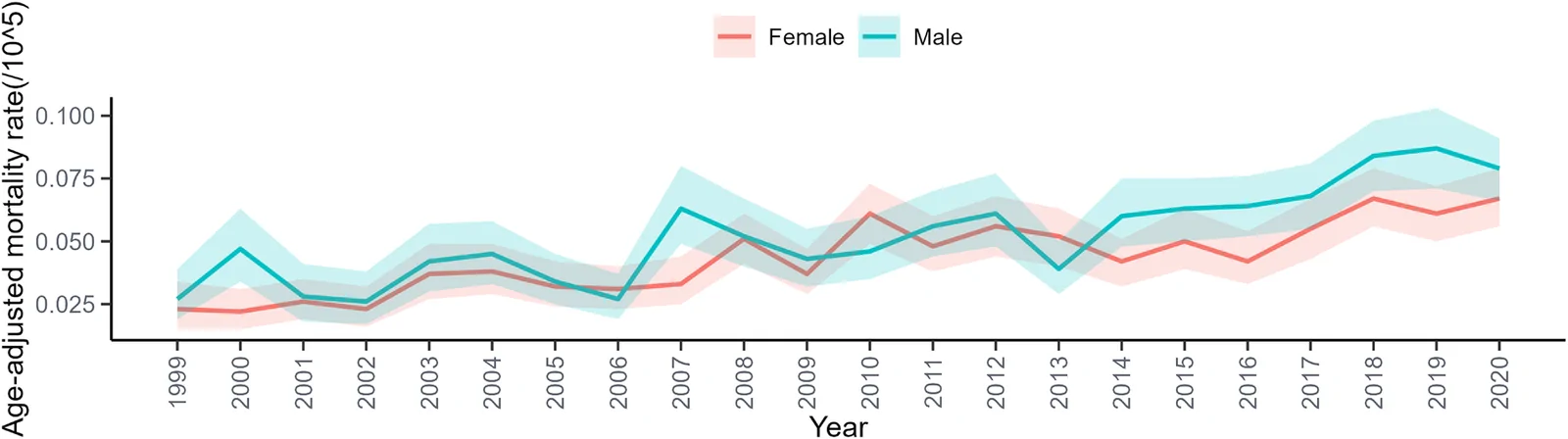
Trends in age-adjusted mortality rates from CVD-listed ADRs by sex in the United States, 1999–2020. AAMR, age-adjusted mortality rate; ADR, adverse drug reaction.

### 3.4 Racial and geographic disparities

Significant racial and geographic disparities were observed in CVD-listed ADR mortality. Among racial groups, Black or African American individuals had the highest AAMR (0.054 per 100,000; 95% CI: 0.048–0.059), followed by American Indian individuals (0.045; 95% CI: 0.029–0.068), while Asian or Pacific Islander individuals had the lowest rate (0.020; 95% CI: 0.015–0.026). White individuals accounted for the largest absolute number of deaths (*n* = 3,319), with an overall AAMR of 0.043 per 100,000 (95% CI: 0.041–0.045). By geographic region, the South contributed the greatest number of deaths (*n* = 1,499), followed by the Midwest (*n* = 917), West (*n* = 840), and Northeast (*n* = 720). In terms of mortality rates, the South had the highest regional AAMR (0.055 per 100,000; 95% CI: 0.052–0.058), while the Midwest and West both showed relatively lower rates (0.045 per 100,000). In 2020, however, regional rates converged within a narrower range, from 0.052 in the Midwest to 0.073 in the Northeast.

### 3.5 Urban–rural disparities

Clear urban–rural disparities were observed, the AAMR of CVD-listed ADR deaths was higher in rural than in urban areas (0.063 vs. 0.042 per 100,000 population). This difference persisted in both 1999 and 2020, with rural rates remaining higher than urban rates in the latter period (0.106 vs. 0.072 per 100,000 in 2020).

### 3.6 Drug-specific findings

Drug-specific descriptive analyses showed that “drug or medicament, unspecified” was recorded in 1,398 deaths (35.2%), indicating that a specific drug category could not be determined for more than one-third of the included deaths (Table 3). Anticoagulants were the most frequently recorded specified drug category, appearing in 948 deaths (23.8% of all included deaths). Other frequently recorded categories included antineoplastic agents, immunosuppressive agents, opioids and related analgesics, and x-ray contrast media. These results represent frequencies of drug-category documentation on death certificates and should not be interpreted as adjudicated causal attribution.

**Table 3:** Most frequently recorded drug categories among CVD-listed ADR deaths, United States, 1999–2020.

Drug	Deaths	Percent of total death
Drug or medicament, unspecified	1,398	35.161%
Anticoagulants	948	23.843%
Other antineoplastic drugs	124	3.119%
Immunosuppressive agents	123	3.094%
Opioids and related analgesics	113	2.842%
x-ray contrast media	113	2.842%
Androgens and anabolic congeners	111	2.792%
Anaesthetic, unspecified	102	2.565%
Anticoagulant antagonists, vitamin K and other coagulants	84	2.113%
Systemic antibiotic, unspecified	78	1.962%

Subgroup analyses across major CVD categories showed that anticoagulants were the most frequently recorded specified drug category across all three CVD subtypes recorded on the death certificates, although the frequency of recording varied substantially (Supplementary Tables S1–S3). Among death records involving hypertensive disease, unspecified drug codes were recorded most frequently (35.9%), followed by anticoagulant codes (17.9%) and x-ray contrast media codes (4.8%). Similarly, among records involving ischaemic heart disease, unspecified drug codes were recorded most frequently (33.2%), while anticoagulant codes were documented in 20.9% of records. In contrast, among records involving cerebrovascular disease, anticoagulant codes were recorded most frequently (60.7%), followed by unspecified drug codes (16.8%) and thrombolytic-agent codes (3.9%).

## 4 Discussion

This nationwide descriptive analysis of U.S. death certificate data identified a significant upward trend from 1999 to 2020 in the age-adjusted mortality rate of deaths in which both an ADR-related code (Y40–Y59) and a CVD code (I00–I99) were recorded. The mortality burden was concentrated among older adults, particularly those aged ≥75 years, and higher rates were observed among males, Black individuals, and rural residents. Unspecified drug codes were recorded most frequently overall; among records containing a specified drug category, anticoagulant codes were the most frequently documented. Because these findings are based on death certificate coding without linkage to medical records, the observed drug-specific patterns reflect frequencies of documentation rather than validated causal drug–death relationships.

A striking finding of this study was the pronounced age gradient in CVD-listed ADR mortality. Those aged 75–84 years and ≥85 years accounted for the majority of deaths, while younger age groups contributed minimally. Older adults with CVD often present with multimorbidity, frailty, reduced physiological reserve, and age-related changes in pharmacokinetics and pharmacodynamics, all of which heighten susceptibility to drug toxicity (13). Moreover, polypharmacy is especially prevalent among older adults with CVD, increasing the likelihood of drug–drug and drug–disease interactions, prescribing cascades, and cumulative treatment burden (14). Reviews in geriatric pharmacology and cardiovascular therapeutics consistently highlight that older CVD patients are particularly vulnerable to ADRs due to the combination of high medication exposure, altered drug handling, and underrepresentation in clinical trials (5). Our age-specific findings therefore emphasize the need for age-sensitive prescribing, judicious deprescribing, and closer monitoring of older adults on complex cardiovascular regimens.

Additionally, this study observed persistently higher mortality rates in men than in women. This finding requires careful consideration, as sex differences in ADRs are multifaceted. A recent systematic review concluded that while sex-related differences in ADR occurrence are common, their direction and magnitude vary significantly by drug class and outcome type (15). Women may experience more frequent ADRs with certain medications, yet men may still have higher fatal ADR-related mortality in populations with substantial baseline cardiovascular risk (16, 17). In the present study, the male excess in mortality could reflect multiple interacting factors, such as a higher burden of severe CVD phenotypes, a greater prevalence of certain behavioral and cardiometabolic risk factors, and poorer long-term cardiovascular outcomes in older men (18). Cohort evidence supports this interpretation, showing that older men tend to experience worse long-term major adverse cardiovascular outcomes than women after myocardial infarction, even when evidence-based therapies are used (19). Thus, in a CVD-focused mortality analysis, the higher fatal ADR burden in men may be attributed to multiple unmeasured factors, including differences in CVD burden, medication exposure, prescribing indications, comorbidities, treatment intensity, fatality following an ADR, and death certification practices. More broadly, cardiovascular risk is increasingly understood to arise from the complex interplay of traditional risk factors, such as hypertension, dyslipidemia, smoking, and obesity, together with emerging inflammatory, metabolic, environmental, and psychosocial determinants (20). Because CDC WONDER lacks medication-exposure denominators and individual-level clinical information, the mechanisms underlying this difference could not be determined. This descriptive finding is therefore hypothesis-generating, and future studies linking mortality records with prescribing data and electronic health records are needed to examine sex-specific risks for individual drug categories.

The racial and urban–rural differences observed in this study are particularly noteworthy. Black individuals had the highest AAMR, and rural residents consistently faced a greater mortality burden than urban residents. A systematic review of racial and ethnic disparities in adverse drug events revealed significant inequities, particularly for high-risk drug classes such as anticoagulants, diabetes agents, and opioids (21). Racial and ethnic minority patients are less likely to initiate anticoagulation or direct oral anticoagulants in certain clinical settings, potentially contributing to disparities in medication safety outcomes (22, 23). Similarly, rural populations face persistent barriers to healthcare access, preventive services, specialist care, and chronic disease management, with national data continuing to show poorer cardiovascular health profiles in rural areas compared to urban populations (24). The observed racial and urban–rural differences may also reflect variation in CVD subtype and severity, comorbidity burden, medication exposure, prescribing intensity, and death certification practices. Because CDC WONDER lacks patient-level clinical and prescribing information, we could not determine the relative contribution of these factors or establish that the observed patterns resulted from inequitable medication safety.

The drug-specific findings provide descriptive signals that may help prioritize questions for future clinically adjudicated research, but they should not be interpreted as direct evidence for drug-specific clinical interventions. Among records with a specified drug category, anticoagulants were the most frequently documented category, particularly among decedents with cerebrovascular disease. This pattern is broadly consistent with previous clinical surveillance studies showing that anticoagulants are commonly involved in serious adverse drug events. The landmark national surveillance study by Budnitz et al. identified anticoagulants as one of the most common causes of emergency hospitalization for adverse drug events in older Americans, particularly due to hemorrhagic complications (25). However, their study examined hospitalization rather than mortality. At the policy level, the U.S. Department of Health and Human Services National Action Plan for Adverse Drug Event Prevention has prioritized anticoagulants, diabetes agents, and opioids due to their substantial, clinically significant, measurable, and largely preventable harm (26). The frequent documentation of anticoagulants among CVD-listed ADR deaths supports continued attention to anticoagulant safety in cardiovascular pharmacovigilance, although clinically linked studies are needed to determine the associated risks among patients with stroke, multimorbidity, advanced age, or complex antithrombotic regimens.

The prominence of other drug categories in our analysis also warrants attention. Antineoplastic drugs and immunosuppressive agents were frequently recorded drug categories, reflecting the increasing intersection of cardiovascular medicine, oncology, and transplant care. Contemporary cardio-oncology guidelines acknowledge that cancer therapies can cause a wide range of cardiovascular toxicities, including cancer therapy-related cardiac dysfunction or heart failure, arrhythmias, ischemia, and thrombotic or vascular complications (27, 28). Similarly, reviews of immunosuppressive therapies have documented significant cardiovascular effects, such as hypertension, dyslipidemia, vascular remodeling, and heightened cardiovascular event risk (29). These mechanisms can be particularly detrimental in death records that also listed CVD. Furthermore, x-ray contrast media were also among the leading recorded drug categories in the included death records. While severe reactions are rare, recent reviews suggest that intravascular contrast media can induce rare but severe hypersensitivity reactions, including Kounis syndrome—an acute coronary hypersensitivity disorder—which can be particularly hazardous for patients with underlying CVD (30, 31). These findings suggest that the future clinically linked studies of fatal ADRs should extend beyond traditional cardiac drugs and promote closer collaboration across cardiology, oncology, radiology, and transplant care.

Another noteworthy aspect of our results is the high proportion of deaths coded as “drug or medicament, unspecified.” This likely reflects a limitation inherent in death certificate-based surveillance rather than an actual absence of drug-related causation. Mortality databases like CDC WONDER are valuable for identifying long-term national patterns in drug-related mortality, but because they rely on death certificate data rather than drug-specific case adjudication, they are less suited than clinical registries or spontaneous reporting systems for assigning specific offending agents (32, 33). As a result, the true contribution of individual drugs may be underestimated or obscured, particularly in CVD patients who often receive multiple concomitant therapies. Nevertheless, the consistent identification of anticoagulants, despite this limitation, strengthens the assertion that they are frequently documented specified drug category in this population. Future research linking death certificate data with claims, electronic health records, or pharmacovigilance databases could help improve causal attribution and identify modifiable intervention points.

Overall, our findings suggest that CVD-listed ADR mortality may reflect a combination of medication exposure, population characteristics, healthcare access, underlying disease burden, and death certification practices. Several non-causal and surveillance-related factors may have contributed to the observed upward trend. The observed upward trend should not be interpreted as demonstrating a corresponding increase in clinically confirmed fatal ADRs. It may partly reflect changes in ADR recognition, death certificate completion, certifier awareness, selection of the underlying cause of death, and application of ICD-10 codes Y40–Y59 over time. Changes in medication exposure, polypharmacy, healthcare delivery, diagnostic investigation, and autopsy practices may also have contributed. These potential explanations could not be distinguished using the available data; therefore, the trend should be interpreted as a descriptive surveillance signal rather than evidence of a causal increase in fatal ADRs.

The study period ended in 2020, which coincided with the onset of the COVID-19 pandemic. Pandemic-related disruptions may have affected death certification practices, medication use, access to healthcare and medication monitoring, and healthcare-seeking behavior. Therefore, the mortality estimate for 2020 may reflect both underlying temporal patterns and pandemic-related influences and should be interpreted cautiously. Moreover, trends after 2020 were not captured in the present analysis, and future studies using more recent CDC WONDER data are warranted.

This study has several limitations. First, as it was based on CDC WONDER mortality data derived from death certificates, the findings are susceptible to inaccuracies in cause-of-death reporting and certification, which may introduce misclassification bias. Race-related estimates should be interpreted with caution, as misclassification on death certificates is a known issue in U.S. mortality data. Second, although ICD-10 codes Y40–Y59 identify deaths in which an adverse drug effect during therapeutic use was assigned as the underlying cause, they encompass heterogeneous mechanisms, including dose-related toxicity during intended treatment, hypersensitivity, idiosyncratic reactions, drug–drug interactions, and drug–disease interactions. Death certificate data do not provide detailed clinical information regarding dose, treatment duration, indication, severity, timing, comorbidities, laboratory findings, or concomitant medications. Consequently, these mechanisms could not be distinguished, and the specific clinical circumstances underlying the recorded deaths could not be determined. Third, the observational and descriptive nature of this study precludes causal inference, and the observed disparities by sex, race, region, and urbanization may reflect unmeasured differences in healthcare access, prescribing patterns, disease severity, and socioeconomic context. Fourth, more than one-third of the included deaths were assigned an unspecified drug code, indicating substantial uncertainty regarding the identity of the drug associated with the recorded adverse effect. Even when a specific drug category was documented, the available data could not determine whether the drug was the proximate cause of death, a contributing factor in the presence of severe comorbidities or polypharmacy, or merely recorded on the death certificate without a clinically adjudicated causal relationship. Finally, as with other mortality-based surveillance studies, the suppression of small cell counts in CDC WONDER may have reduced precision in certain subgroup analyses, particularly among smaller racial groups. Despite these limitations, the database remains a valuable resource for identifying long-term national patterns and disparities in fatal ADR-related mortality.

## 5 Conclusion

In this descriptive analysis of U.S. death certificate data, CVD-listed ADR mortality increased from 1999 to 2020, with differences observed across age, sex, race, and urbanization groups. Unspecified drugs accounted for more than one-third of the included deaths, and anticoagulants were the most frequently recorded specified drug category. Because the data lacked clinical adjudication and linkage to medical or prescribing records, these findings do not establish drug-specific causality or identify the mechanisms underlying the observed demographic and geographic differences. The results therefore require confirmation in studies incorporating electronic health records, claims data, medication exposure information, and case-level clinical review.

## Data Availability

The data analyzed in this study is subject to the following licenses/restrictions: the datasets used and analyzed during this study are available from the corresponding author upon reasonable request. Requests to access these datasets should be directed to Hong Zhu, hayyzhuhong@163.com.
