# Supplementary material for: Mortality trends in deaths attributed to adverse drug reactions with cardiovascular disease listed among multiple causes in the United States, 1999–2020
Source: Front Cardiovasc Med. 2026 Oct 1;13:1857514. doi: 10.3389/fcvm.2026.1857514 (PMC13668594; doi:10.3389/fcvm.2026.1857514)
Supplement: Supplementary file 1 [file Datasheet1.docx]

Table S1. Top 10 most frequently recorded drug categories with hypertensive disease–listed ADR deaths, United States, 1999–2020

| Drug | Deaths | Percent of Total Death |
| --- | --- | --- |
| Drug or medicament, unspecified | 285 | 35.939% |
| Anticoagulants | 142 | 17.907% |
| X-ray contrast media | 38 | 4.792% |
| Angiotensin-converting-enzyme inhibitors | 32 | 4.035% |
| Androgens and anabolic congeners | 28 | 3.531% |
| Immunosuppressive agents | 24 | 3.026% |
| Opioids and related analgesics | 22 | 2.774% |
| Systemic antibiotic, unspecified | 21 | 2.648% |
| Anaesthetic, unspecified | 21 | 2.648% |
| Other antineoplastic drugs | 17 | 2.144% |

Table S2. Top 10 most frequently recorded drug categories with ischaemic heart diseases–listed ADR deaths, United States, 1999–2020

| Drug | Deaths | Percent of Total Death |
| --- | --- | --- |
| Drug or medicament, unspecified | 321 | 33.230% |
| Anticoagulants | 202 | 20.911% |
| X-ray contrast media | 46 | 4.762% |
| Anticoagulant antagonists, vitamin K and other coagulants | 44 | 4.555% |
| Androgens and anabolic congeners | 29 | 3.002% |
| Opioids and related analgesics | 29 | 3.002% |
| Systemic antibiotic, unspecified | 24 | 2.484% |
| Immunosuppressive agents | 23 | 2.381% |
| Insulin and oral hypoglycaemic [antidiabetic] drugs | 22 | 2.277% |
| Other antineoplastic drugs | 21 | 2.174% |

Table S3. The most frequently recorded drug categories with Cerebrovascular diseases–listed ADR deaths, United States, 1999–2020

| Drug | Deaths | Percent of Total Death |
| --- | --- | --- |
| Anticoagulants | 390 | 60.653% |
| Drug or medicament, unspecified | 108 | 16.796% |
| Thrombolytic drugs | 25 | 3.888% |

Supplementary Table S4. ICD-10 Codes Y40–Y59: Drugs, Medicaments, and Biological Substances Causing Adverse Effects in Therapeutic Use

| **ICD-10 code** | **Category or detailed title** |
| --- | --- |
| **Y40** | **Systemic antibiotics** |
| Y40.0 | Penicillins |
| Y40.1 | Cephalosporins and other beta-lactam antibiotics |
| Y40.2 | Chloramphenicol group |
| Y40.3 | Macrolides |
| Y40.4 | Tetracyclines |
| Y40.5 | Aminoglycosides |
| Y40.6 | Rifamycins |
| Y40.7 | Antifungal antibiotics, systemically used |
| Y40.8 | Other systemic antibiotics |
| Y40.9 | Systemic antibiotic, unspecified |
| **Y41** | **Other systemic anti-infectives and antiparasitics** |
| Y41.0 | Sulfonamides |
| Y41.1 | Antimycobacterial drugs |
| Y41.2 | Antimalarials and drugs acting on other blood protozoa |
| Y41.3 | Other antiprotozoal drugs |
| Y41.4 | Anthelminthics |
| Y41.5 | Antiviral drugs |
| Y41.8 | Other specified systemic anti-infectives and antiparasitics |
| Y41.9 | Systemic anti-infective and antiparasitic, unspecified |
| **Y42** | **Hormones and their synthetic substitutes and antagonists, not elsewhere classified** |
| Y42.0 | Glucocorticoids and synthetic analogues |
| Y42.1 | Thyroid hormones and substitutes |
| Y42.2 | Antithyroid drugs |
| Y42.3 | Insulin and oral hypoglycaemic [antidiabetic] drugs |
| Y42.4 | Oral contraceptives |
| Y42.5 | Other estrogens and progestogens |
| Y42.6 | Antigonadotrophins, antiestrogens, antiandrogens, not elsewhere classified |
| Y42.7 | Androgens and anabolic congeners |
| Y42.8 | Other and unspecified hormones and their synthetic substitutes |
| Y42.9 | Other and unspecified hormone antagonists |
| **Y43** | **Primarily systemic agents** |
| Y43.0 | Antiallergic and antiemetic drugs |
| Y43.1 | Antineoplastic antimetabolites |
| Y43.2 | Antineoplastic natural products |
| Y43.3 | Other antineoplastic drugs |
| Y43.4 | Immunosuppressive agents |
| Y43.5 | Acidifying and alkalizing agents |
| Y43.6 | Enzymes, not elsewhere classified |
| Y43.8 | Other primarily systemic agents, not elsewhere classified |
| Y43.9 | Primarily systemic agent, unspecified |
| **Y44** | **Agents primarily affecting blood constituents** |
| Y44.0 | Iron preparations and other anti-hypochromic-anemia preparations |
| Y44.1 | Vitamin B12, folic acid, and other anti-megaloblastic-anemia preparations |
| Y44.2 | Anticoagulants |
| Y44.3 | Anticoagulant antagonists, vitamin K, and other coagulants |
| Y44.4 | Antithrombotic drugs [platelet-aggregation inhibitors] |
| Y44.5 | Thrombolytic drugs |
| Y44.6 | Natural blood and blood products |
| Y44.7 | Plasma substitutes |
| Y44.9 | Other and unspecified agents affecting blood constituents |
| **Y45** | **Analgesics, antipyretics, and anti-inflammatory drugs** |
| Y45.0 | Opioids and related analgesics |
| Y45.1 | Salicylates |
| Y45.2 | Propionic acid derivatives |
| Y45.3 | Other nonsteroidal anti-inflammatory drugs [NSAID] |
| Y45.4 | Antirheumatics |
| Y45.5 | 4-Aminophenol derivatives |
| Y45.8 | Other analgesics, antipyretics, and anti-inflammatory drugs |
| Y45.9 | Analgesic, antipyretic, and anti-inflammatory drug, unspecified |
| **Y46** | **Antiepileptics and antiparkinsonism drugs** |
| Y46.0 | Succinimides |
| Y46.1 | Oxazolidinediones |
| Y46.2 | Hydantoin derivatives |
| Y46.3 | Deoxybarbiturates |
| Y46.4 | Iminostilbenes |
| Y46.5 | Valproic acid |
| Y46.6 | Other and unspecified antiepileptics |
| Y46.7 | Antiparkinsonism drugs |
| Y46.8 | Antispasticity drugs |
| **Y47** | **Sedatives, hypnotics, and antianxiety drugs** |
| Y47.0 | Barbiturates, not elsewhere classified |
| Y47.1 | Benzodiazepines |
| Y47.2 | Chloral derivatives |
| Y47.3 | Paraldehyde |
| Y47.4 | Bromine compounds |
| Y47.5 | Mixed sedatives and hypnotics, not elsewhere classified |
| Y47.8 | Other sedatives, hypnotics, and antianxiety drugs |
| Y47.9 | Sedative, hypnotic, and antianxiety drug, unspecified |
| **Y48** | **Anesthetics and therapeutic gases** |
| Y48.0 | Inhaled anesthetics |
| Y48.1 | Parenteral anesthetics |
| Y48.2 | Other and unspecified general anesthetics |
| Y48.3 | Local anesthetics |
| Y48.4 | Anesthetic, unspecified |
| Y48.5 | Therapeutic gases |
| **Y49** | **Psychotropic drugs, not elsewhere classified** |
| Y49.0 | Tricyclic and tetracyclic antidepressants |
| Y49.1 | Monoamine-oxidase-inhibitor antidepressants |
| Y49.2 | Other and unspecified antidepressants |
| Y49.3 | Phenothiazine antipsychotics and neuroleptics |
| Y49.4 | Butyrophenone and thioxanthene neuroleptics |
| Y49.5 | Other antipsychotics and neuroleptics |
| Y49.6 | Psychodysleptics [hallucinogens] |
| Y49.7 | Psychostimulants with abuse potential |
| Y49.8 | Other psychotropic drugs, not elsewhere classified |
| Y49.9 | Psychotropic drug, unspecified |
| **Y50** | **Central nervous system stimulants, not elsewhere classified** |
| Y50.0 | Analeptics |
| Y50.1 | Opioid receptor antagonists |
| Y50.2 | Methylxanthines, not elsewhere classified |
| Y50.8 | Other central nervous system stimulants |
| Y50.9 | Central nervous system stimulant, unspecified |
| **Y51** | **Drugs primarily affecting the autonomic nervous system** |
| Y51.0 | Anticholinesterase agents |
| Y51.1 | Other parasympathomimetics [cholinergics] |
| Y51.2 | Ganglionic blocking drugs, not elsewhere classified |
| Y51.3 | Other parasympatholytics [anticholinergics and antimuscarinics] and spasmolytics, not elsewhere classified |
| Y51.4 | Predominantly alpha-adrenoreceptor agonists, not elsewhere classified |
| Y51.5 | Predominantly beta-adrenoreceptor agonists, not elsewhere classified |
| Y51.6 | Alpha-adrenoreceptor antagonists, not elsewhere classified |
| Y51.7 | Beta-adrenoreceptor antagonists, not elsewhere classified |
| Y51.8 | Centrally acting and adrenergic-neuron-blocking agents, not elsewhere classified |
| Y51.9 | Other and unspecified drugs primarily affecting the autonomic nervous system |
| **Y52** | **Agents primarily affecting the cardiovascular system** |
| Y52.0 | Cardiac-stimulant glycosides and drugs of similar action |
| Y52.1 | Calcium-channel blockers |
| Y52.2 | Other antidysrhythmic drugs, not elsewhere classified |
| Y52.3 | Coronary vasodilators, not elsewhere classified |
| Y52.4 | Angiotensin-converting-enzyme inhibitors |
| Y52.5 | Other antihypertensive drugs, not elsewhere classified |
| Y52.6 | Antihyperlipidemic and antiarteriosclerotic drugs |
| Y52.7 | Peripheral vasodilators |
| Y52.8 | Antivaricose drugs, including sclerosing agents |
| Y52.9 | Other and unspecified agents primarily affecting the cardiovascular system |
| **Y53** | **Agents primarily affecting the gastrointestinal system** |
| Y53.0 | Histamine H2-receptor antagonists |
| Y53.1 | Other antacids and anti-gastric-secretion drugs |
| Y53.2 | Stimulant laxatives |
| Y53.3 | Saline and osmotic laxatives |
| Y53.4 | Other laxatives |
| Y53.5 | Digestants |
| Y53.6 | Antidiarrheal drugs |
| Y53.7 | Emetics |
| Y53.8 | Other agents primarily affecting the gastrointestinal system |
| Y53.9 | Agent primarily affecting the gastrointestinal system, unspecified |
| **Y54** | **Agents primarily affecting water-balance and mineral and uric acid metabolism** |
| Y54.0 | Mineralocorticoids |
| Y54.1 | Mineralocorticoid antagonists [aldosterone antagonists] |
| Y54.2 | Carbonic-anhydrase inhibitors |
| Y54.3 | Benzothiadiazine derivatives |
| Y54.4 | Loop [high-ceiling] diuretics |
| Y54.5 | Other diuretics |
| Y54.6 | Electrolytic, caloric, and water-balance agents |
| Y54.7 | Agents affecting calcification |
| Y54.8 | Agents affecting uric acid metabolism |
| Y54.9 | Mineral salts, not elsewhere classified |
| **Y55** | **Agents primarily acting on smooth and skeletal muscles and the respiratory system** |
| Y55.0 | Oxytocic drugs |
| Y55.1 | Skeletal muscle relaxants [neuromuscular blocking agents] |
| Y55.2 | Other and unspecified agents primarily acting on muscles |
| Y55.3 | Antitussives |
| Y55.4 | Expectorants |
| Y55.5 | Anticommon-cold drugs |
| Y55.6 | Antiasthmatics, not elsewhere classified |
| Y55.7 | Other and unspecified agents primarily acting on the respiratory system |
| **Y56** | **Topical agents primarily affecting skin and mucous membrane and ophthalmological, otorhinolaryngological, and dental drugs** |
| Y56.0 | Local antifungal, anti-infective, and anti-inflammatory drugs, not elsewhere classified |
| Y56.1 | Antipruritics |
| Y56.2 | Local astringents and local detergents |
| Y56.3 | Emollients, demulcents, and protectants |
| Y56.4 | Keratolytics, keratoplastics, and other hair treatment drugs and preparations |
| Y56.5 | Ophthalmological drugs and preparations |
| Y56.6 | Otorhinolaryngological drugs and preparations |
| Y56.7 | Dental drugs, topically applied |
| Y56.8 | Other topical agents |
| Y56.9 | Topical agent, unspecified |
| **Y57** | **Other and unspecified drugs and medicaments** |
| Y57.0 | Appetite depressants [anorectics] |
| Y57.1 | Lipotropic drugs |
| Y57.2 | Antidotes and chelating agents, not elsewhere classified |
| Y57.3 | Alcohol deterrents |
| Y57.4 | Pharmaceutical excipients |
| Y57.5 | X-ray contrast media |
| Y57.6 | Other diagnostic agents |
| Y57.7 | Vitamins, not elsewhere classified |
| Y57.8 | Other drugs and medicaments |
| Y57.9 | Drug or medicament, unspecified |
| **Y58** | **Bacterial vaccines** |
| Y58.0 | BCG vaccine |
| Y58.1 | Typhoid and paratyphoid vaccine |
| Y58.2 | Cholera vaccine |
| Y58.3 | Plague vaccine |
| Y58.4 | Tetanus vaccine |
| Y58.5 | Diphtheria vaccine |
| Y58.6 | Pertussis vaccine, including combinations with a pertussis component |
| Y58.8 | Mixed bacterial vaccines, except combinations with a pertussis component |
| Y58.9 | Other and unspecified bacterial vaccines |
| **Y59** | **Other and unspecified vaccines and biological substances** |
| Y59.0 | Viral vaccines |
| Y59.1 | Rickettsial vaccines |
| Y59.2 | Protozoal vaccines |
| Y59.3 | Immunoglobulin |
| Y59.8 | Other specified vaccines and biological substances |
| Y59.9 | Vaccine or biological substance, unspecified |
